# Assessment of metered-dose inhaler technique: A study at the pulmonology clinic of a tertiary hospital in the Free State, South Africa

**DOI:** 10.7196/AJTCCM.2019.v25i1.232

**Published:** 2019-04-12

**Authors:** Y Ramkillawan, M Prins, C van van Rooyen, R Y Seedat

**Affiliations:** 1 Department of Internal Medicine, Faculty of Health Sciences, University of the Free State and Universitas Academic Hospital, Bloemfontein, South Africa; 2 Department of Biostatistics, Faculty of Health Sciences, University of the Free State, Bloemfontein, South Africa; 3 Department of Otorhinolaryngology, Faculty of Health Sciences, University of the Free State and Universitas Academic Hospital, Bloemfontein, South Africa

**Keywords:** inhalers, technique, metered dose inhaler, spacers

## Abstract

**Background:**

Poor pressurised metered-dose inhaler (pMDI) technique remains a challenge in the management of airway diseases.

**Objectives:**

To assess pMDI technique among respiratory outpatients and identify the main indications for pMDI use and factors associated with improper use.

**Methods:**

This was a prospective, quantitative descriptive study conducted at the adult respiratory clinic of Universitas Academic Hospital in Bloemfontein, South Africa. A convenience sample of 100 participants was used. Each participant was interviewed and required to demonstrate the use of a placebo pMDI, either alone or with a large-volume spacer. Inhaler technique was evaluated according to the UK Inhaler Group standard for inhaler therapy.

**Results:**

Chronic obstructive pulmonary disease and asthma were the most common indications for pMDI use. Of the 100 participants, 97 preferred a pMDI without a spacer (pMDI alone) and three preferred using the inhaler with a spacer. In the pMDI-alone group, 13 participants (13.4%) demonstrated correct technique and 65 (67%) made more than one error.

**Conclusion:**

Poor inhaler technique is common among respiratory outpatients. Every contact with the patient should be an opportunity to reinforce correct pMDI technique.

## Background

Chronic lower respiratory tract disease is the eighth leading cause of mortality in South Africa (SA).^[1]^ Inhaled therapy is the mainstay of managing most airway diseases.^[2]^ It has the advantage that the drug is delivered directly to the site of need, which means a lower dose can be used to achieve the same effect as another preparation, and it has a reduced side-effect profile compared with other routes of administration.^[3]^ Once the medication has been inhaled, the respiratory tract uses both active and passive transport mechanisms to facilitate absorption from the epithelial surface and transfer to the rest of the tracheobronchial tree.^[3]^

Despite the advantages of inhaled therapy, a major limiting factor in appropriate drug delivery is incorrect technique in using a pressurised metered-dose inhaler (pMDI). Improper technique has been associated with frequent visits to the emergency department, adding to the economic burden of hospitalisations, poor disease control and poor quality of life.^[4]^ The improper use of pMDIs has been demonstrated in SA^[5,6]^ and also in several studies globally.^[4,7-13]^ Studies seeking to investigate causal factors for improper pMDI technique have shown that both patient factors (age, race, gender, education) and patient preferences contribute to the outcome.^[2,11,14]^ However, results are inconsistent and no clear predictors for profiling patients with improper technique have emerged.

Although accuhalers are available at our setting (a tertiary-level hospital pharmacy), only pMDIs are available via the Department of Health at the regional and district pharmacies in our province (Free State). Other SA studies evaluating pMDI technique have been conducted in private practice and rural clinics. We evaluated pMDI technique among the outpatients in our tertiary hospital clinic. We also evaluated the reasons for pMDI use and described factors associated with poor technique.

## Methods

### Study design and participants

This was a prospective, quantitative, descriptive study conducted at the respiratory clinic of the Universitas Academic Hospital in Bloemfontein, Free State. In this study, we assessed pMDI technique among adult respiratory outpatients. We also determined the main indications for pMDI use at our clinic and attempted to identify factors associated with improper use. All adult patients using at least one pMDI were invited to participate in the study, irrespective of their medical diagnosis. A convenience sample of 100 patients was used.

The study was conducted according to the principles of the Helsinki Declaration and written informed consent was obtained from all participants. Ethical approval was obtained from the Health Sciences Research Ethics Committee at the University of the Free State (ref. no. UFS-HSD2017/0435) and the Free State Department of Health.

### Data collection

Participants were interviewed by the principal investigator to obtain sociodemographic and clinical data and perceptions about their inhaler. The interview was conducted in English or Afrikaans, based on the participant’s language preference. After the interview, participants were asked to demonstrate their inhaler technique, using either a placebo pMDI alone or a placebo pMDI coupled with a large-volume spacer. The inhaler technique was evaluated by observation, using a checklist aligned with the UK Inhaler Group’s standard for inhaler therapy (Tables 1 and 2).^[15]^ Incorrect technique was defined as having performed any of the steps incorrectly and hence achieving a score <7, irrespective of whether the pMDI was used alone or with a spacer. The study questionnaire was pretested on five participants in a pilot study to assess participant comprehension of the questions.

**Table 1:** Checklist for use of a pressurised metered-dose inhaler alone*

1. Remove the mouthpiece cover.
2. Shake the inhaler.
3. Breathe out as far as is comfortable.
4. Place inhaler in mouth and close your lips around it.
5. As you breathe in press the canister down and continue breathing in slow and steady.
6. Remove device from mouth and hold breath for up to 10 seconds.
7. Wait for a few seconds before repeating the dose and repeat the process if needed. Then replace the mouthpiece cover.

*Phrasing as per the UK Inhaler Group standard for inhaler therapy.^[15]^

**Table 2:** Checklist for using a pressurised metered-dose inhaler with a spacer*

1. Remove cap and shake the inhaler
2. Insert inhaler into spacer through the hole at the end
3. Breathe out gently as far as is comfortable
4. Place spacer mouthpiece in mouth and close lips around it
5. Press canister down and breathe in deeply (or tidal breath, several breaths in and out). If the device whistles your breath is too fast (small spacer).
6. Remove from mouth and hold breath for up to 10 seconds.
7. Wait a few seconds and repeat process if needed.

*Phrasing as per the UK Inhaler Group standard for inhaler therapy.^[15]^

### Statistical analysis

Descriptive statistics were used to summarise participants’ sociodemographic and clinical characteristics. Categorical variables were described using absolute frequencies and percentages. Continuous variables were described as a mean with standard deviation or a median with interquartile range (IQR), as appropriate for the data distribution. Comparisons were performed using Pearson’s chi-squared test.

## Results

### Baseline demographics and clinical characteristics

There was female predominance in the study population (56%) and the median (IQR) age of participants was 59 (46.5 - 66) years. Participants had been reviewed at the clinic for a median (IQR) of 51 (11.5 - 74.5) months. Two-thirds of participants (67%) were referred from surrounding hospitals and the others were referred either from a general practitioner (24%) or a nearby clinic (9%). All of the participants were literate in Afrikaans (78%) or English (66%). The participants who were literate in Sesotho (42%) were also literate in Afrikaans. A large number of participants had not completed school (59%). The remainder was made up of 27 participants (27%) who had completed school but did not have a tertiary qualification and 14 participants (14%) with a subsequent tertiary qualification.

### Indications for inhaler use, medication type and duration of use 

Obstructive lung disease was the most common indication for pMDI use, with chronic obstructive pulmonary disease (COPD) and asthma accounting for 35% and 32% of the diagnoses, respectively. The median (IQR) age of participants with COPD was 64 (57 - 69) years, whereas that of the participants with asthma was 56 (46 - 62) years. Asthma-COPD overlap occurred in one patient. Less common indications for pMDI use were bronchiectasis (17%) and interstitial lung disease (11%). The median (IQR) age of patients with bronchiectasis and interstitial lung disease was 45 (35 - 60) years and 56 (43 - 61) years , respectively. Seven patients had more than one indication for pMDI use.

More than half the participants used three or more inhalers (54%). Salbutamol was the most widely used substance (87%). The profile of pMDI medication types and the duration of use are described in Table 3.

**Table 3:** Type of pressurised metered-dose inhaler and duration of use

**Medication type**	**Number of patients (N=100)**	**Median duration of use (months)**
Salbutamol	87	60
Formoterol	56	42
Ipratropium bromide	44	33
Beclomethasone dipropionate	41	24
Budesonide	22	47
Ipratropium-fenoterol	6	87

### Patient education and perceptions regarding pMDI use

The lung function technologist was the primary source of education about pMDI technique for 43 of the participants. Other sources of education included doctors at the respiratory clinic (39%), primary care nurses (7%) and the attending pharmacist (1%). Ten participants reported their pMDI technique was self-taught.

In 75 participants, pMDI technique was checked within the last 12 months prior to our study. Ten participants had their technique checked 1 year before the study and 9 participants’ technique had last been reviewed between 2 and 23 years ago. Six participants had never had their inhaler technique checked.

Difficulty in using the inhaler adequately was reported by 23 participants. The most common reasons were: forgetting when to use their inhaler (15%); difficulty in holding the inhaler (6%); difficulty in co-ordinating administering the medication and inhaling (3%), and difficulty in holding their breath for at least 10 seconds (2%). Three participants each reported two reasons for finding pMDI use difficult.

### Evaluation of pMDI technique

**Fig. 1:**
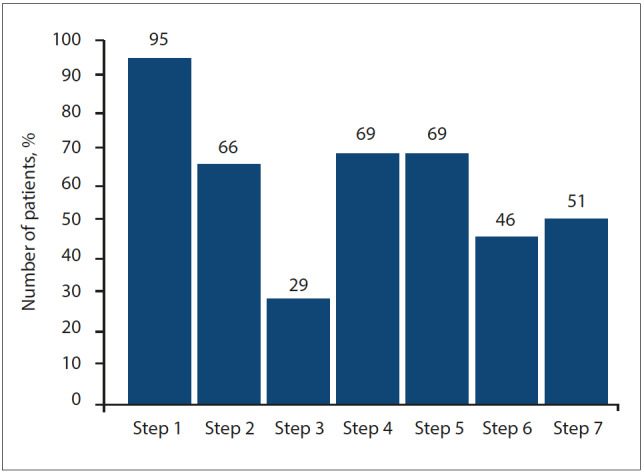
Frequency of correctly performed steps by patients using a pressurised metered-dose inhaler without a spacer (N=97).

The majority (97%) of participants preferred to use a pMDI without a spacer (pMDI alone), with only three participants preferring to use a pMDI with a spacer. Among participants who preferred to use a pMDI without a spacer, only 13 (13.4%) performed all seven steps correctly. The first step (removing the mouthpiece) was most frequently performed correctly in this group (n=95, 98%) (Fig. 1).

**Fig. 2:**
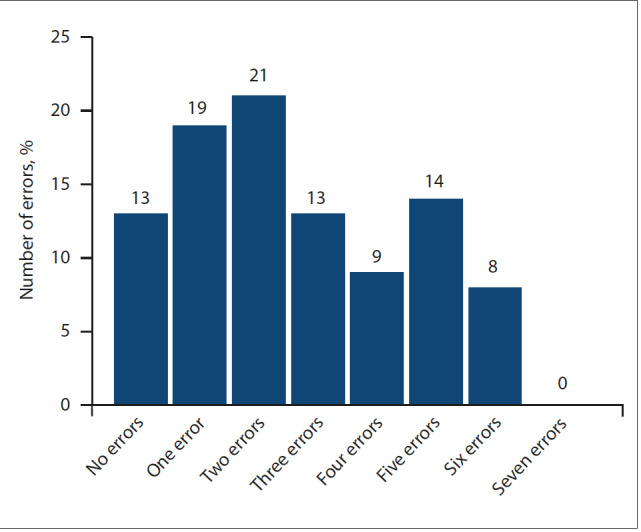
Frequency of errors in inhaler technique (N=97).

Errors in handling the inhaler were observed in 86.6% of the participants who preferred to use it without a spacer, with the most common error being not breathing out prior to placing the device in their mouth (70%). Participants also struggled with removing the inhaler from their mouth while holding their breath for up to 10 seconds (53%), and waiting before repeating the process if needed (47%). Multiple errors in inhaler technique were seen in 65 participants (67%) (Fig. 2).

### Associations with poor inhaler technique 

An education level of lower than Gr. 12 was associated with poor inhaler technique (p=0.01). Neither type of educator (p=0.64) nor participants’ perception of difficulty in using the inhaler (p=0.72) was associated with incorrect technique.

## Discussion

Our study explored pMDI technique in a sample of respiratory outpatients at Universitas Academic Hospital in Bloemfontein, Free State. The objectives of the study were to list the indications for pMDI use and to describe the characteristics of patients and factors associated with incorrect technique. The majority of the patients in this sample could not use their inhaler correctly. The main indications for the use of a pMDI were COPD, asthma and bronchiectasis. A low level of academic education was associated with incorrect inhaler technique.

The median age of COPD patients in this study was 64 years. This is in keeping with other studies where COPD is the main indication for pMDI use.^[7]^ In studies that reported asthma as the main indication for pMDI use, the median age of participants was lower (40.4 - 47 years)^[4,7-9,16]^ than for asthmatic patients in our study. The reason for this difference is unclear, but the median age of participants in our study is a reflection of our study population.

In our study, almost half the participants were educated on inhaler technique by doctors, nurses or pharmacists. Several SA studies have shown training on pMDI technique to be problematic. In a study across seven rural health clinics in SA, only 43% of adults could use their inhaler correctly, despite regular training.^[5]^ An audit of clinics in the Western Cape revealed a considerable lack of placebo pMDIs and spacers in consulting rooms,^[17]^ which suggests that the quality of training offered at clinic level in this province may be compromised. An assessment of pMDI technique among healthcare workers and final-year medical students in Johannesburg showed that only 16% of participants could perform the technique correctly.^[18]^ A similar poor performance was demonstrated among pharmacists in Ethiopia, where only 4.8% of the respondents were competent in using a pMDI.^[19]^ Further studies are needed to assess the reliability of pMDI education among healthcare workers in the Free State, as this may contribute to incorrect technique among patients.

Errors in using a pMDI can occur despite repeat reinforcement and education.^[5,20]^ In a study at a private practice in SA, pMDI technique was inferior to that for dry-powder inhalers and patients using accuhalers performed worse than those on turbohalers.^[6]^ Similar findings were reported in a study by Khassawneh *et al*.^[9]^ None of our participants used dry-powder inhalers. Switching to a different type of device should be considered when technique remains inadequate despite repeat training.

Improper pMDI technique was seen in 86.6% of participants in our study. We found more incorrectly performed steps than other studies in SA.^[5,6]^ Possible reasons for this include differences in study populations, participants’ level of education not being considered and different standards used for comparison.

Our results are comparable with those from international studies, which have demonstrated improper technique in 45% - 95% of patients.^[4,7-12]^ However, our participants showed fewer errors in their technique than those reported in a study by Giraud and Roche.^[11]^ This finding may be due to the smaller sample size in our study. Our patients mostly showed difficulty with breathing out prior to placing the inhaler in their mouth (step 3 in the process) and removing the inhaler from their mouth while holding their breath (step 6). Poor hand-lung co-ordination has been described in several studies and is a potential reason for pMDIs being regarded as difficult to use.^[6,9-11,13]^

There are several possible reasons for the errors in pMDI technique seen in our study. First, a strict criterion was used to define proper inhaler technique. This may overestimate the frequency of errors to some extent. It has previously been suggested that the most important variables are the absolute volume of air inhaled, the inspiratory flow rate and the duration of the end-expiratory breath hold.^[3]^ Inhaling too fast or a too small volume of air may result in particles being deposited in the oropharynx instead of penetrating into the lung.^[3]^ Time allocated to holding the breath allows the particles to settle in the lungs, which contributes to the efficacy of the drug.^[3]^ However, there is no agreement regarding clinically significant critical errors.

Second, despite pMDI technique having been previously checked in 94% of participants, a large number of errors were still observed. It is suggested that repeat instructions can improve pMDI technique.^[20,21]^ In a study by Weinberg and Naya,^[20]^ pMDI technique improved from 32% to 86% by repeatedly reinforcing correct technique. In our study, we did not determine how often pMDI technique was assessed.

Third, a lower level of academic education was associated with poor pMDI technique. This may have contributed to the poor performance in our study. In contrast to other investigators, we found no other factors associated with improper pMDI technique, such as number of devices used, increasing age of participants or duration of inhaler use.^[2,11,14]^ However, our study was not specifically designed to determine associations with poor inhaler technique and further studies are needed to delineate these associations in our setting.

### Study limitations

There were several limitations in our study. We used a small convenience sample, which may limit the generalisability of this finding. Our institution reviews mainly complicated lung diseases and we interviewed only outpatients. This was in an attempt to exclude patients who were too dyspnoeic to use a pMDI. However, referral and selection bias must be considered. 

Participants’ pMDI technique was evaluated immediately after their giving consent and the interview had been conducted. Anxiety around having their technique assessed could have contributed to some errors. In addition, participants were all interviewed by the same investigator. All the questions were answered by the participant and information was not corroborated with medical records. Interviewer bias may therefore limit the generalisability of the finding.

There is a lack of consensus on how to assess pMDI technique. We weighted each step equally although some steps may be regarded as more critical than others; however, there is no agreement on which steps these might be. This factor may have overestimated the occurrence of incorrect technique, as using a comparative standard does not assign a relative importance to each step. Different studies also use different methods to assess improper technique, namely critical errors,^[22]^ rate of wrong steps,^[2]^ essential steps^[9]^ or error cutoffs.[8,11] As these are different methods of assessment, they cannot be accurately compared.

## Conclusion

The role of pMDIs in respiratory medicine is well established. However, inhaler technique can be fraught with difficulty and errors. In this study, we identified that the main indications for pMDI use was COPD and asthma. Incorrect pMDI technique occurred in 86.6% of our sample, with 65 participants (67%) having made more than one error. These findings have important implications for disease management, patients’ quality of life and medical costs. Solutions to addressing these fundamental errors lie in the education of healthcare workers and patients and, if necessary, considering changing the inhaler type. Further research is needed to delineate factors associated with poor pMDI technique.
